# Clinical Complete Remission of An Advanced Gastric Adenocarcinoma After Camrelizumab Plus Chemotherapy Followed by Camrelizumab Plus Capecitabine: A Case Report

**DOI:** 10.3389/fonc.2021.775147

**Published:** 2021-12-22

**Authors:** Jieheng Lin, Jianying Yang, Wenping Wang, Xiaotong Lin, Yang Cao

**Affiliations:** ^1^ Department of Oncology Center, the First Affiliated Hospital of Guangzhou University of Chinese Medicine, Guangzhou, China; ^2^ The First Clinical Medical College of Guangzhou University of Chinese Medicine, Guangzhou, China

**Keywords:** camrelizumab, chemotherapy, advanced gastric adenocarcinoma, clinical complete response (CCR), peritoneal metastasis

## Abstract

We report a rare case of PDL1-negative advanced gastric adenocarcinoma that improved significantly after camrelizumab plus chemotherapy followed by camrelizumab plus capecitabine as first-line therapy. A 65-year-old woman was diagnosed with a gastric adenocarcinoma in 2017 *via* contrast-enhanced computed tomography (CT) and endoscopic biopsy. She stabilised after preoperative neoadjuvant chemotherapy, surgery, and postoperative adjuvant chemotherapy. In September 2019, positron emission tomography (PET)/CT re-examination suggested a peritoneal metastasis and multiple lymph node metastases. She then received six cycles of camrelizumab plus chemotherapy. PET/CT indicated that the metastatic foci had disappeared and that she had achieved a clinical complete response(CCR). She was followed-up with camrelizumab plus capecitabine (maintenance therapy). At the time of writing, her progression-free survival is more than 14 months and her quality of life is good. Thus, camrelizumab plus chemotherapy is a useful first-line treatment for HER2- and PD-L1-negative advanced gastric adenocarcinoma.

## Introduction

Gastric cancer is the fifth most common cancer worldwide and the fourth leading cause of cancer-related death (1). Peritoneal metastasis is the most common form of recurrence and the second-most important cause of death from advanced gastric cancer (after radical surgery). Peritoneal metastasis is caused by haematogenous, lymphatic, or direct peritoneal implantation of primary gastric cancer cells (2). Almost 20% of gastric cancer patients are diagnosed with peritoneal metastases before or during surgery, and over 50% of stage T3 and T4 patients develop such metastases after radical resection. The higher the grade of peritoneal metastasis, the shorter the survival (3). In China, 53–60% of patients with gastric cancer metastases have peritoneal metastases; the 5-year survival rate of such patients is only 2% and the median survival time only 3–6 months (4).

Systemic chemotherapy is an effective first-line treatment for advanced gastric adenocarcinoma. Currently, two drug regimens featuring fluorouracil combined with platinum or taxanes are used in clinical practice. However, the effectiveness of chemotherapy alone is limited. In patients with advanced gastric adenocarcinomas, immunotherapy combined with chemotherapy affords stronger anti-tumor activity as first-line therapy than does chemotherapy alone (5, 6). In our present case, the patient achieved a clinical complete response (CCR) after six cycles of treatment with carrizumab plus capecitabine, and her progression-free survival (PFS) is currently 14 months on maintenance treatment with carrizumab plus capecitabine. Such treatment may be useful for patients with advanced gastric adenocarcinomas.

## Case Report

**Figure 1:**
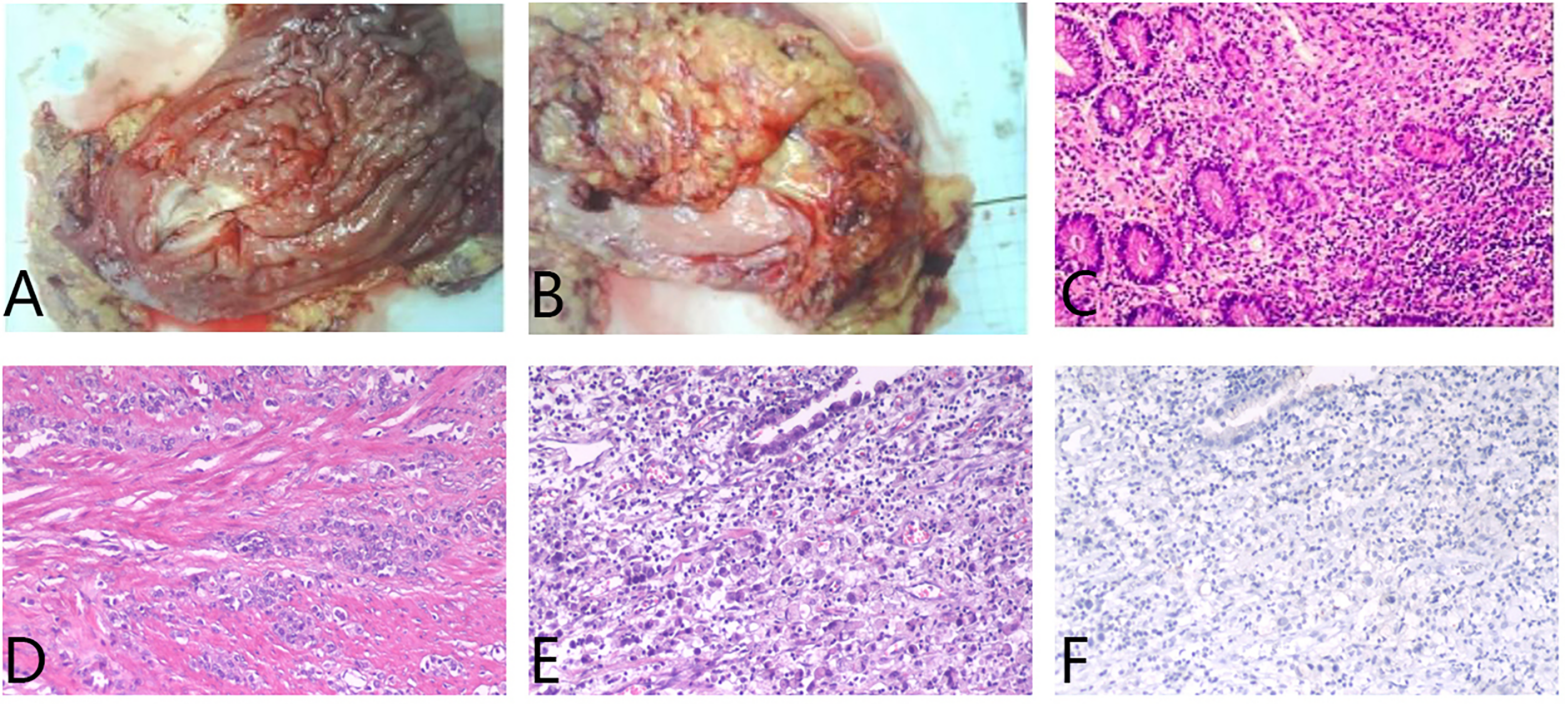
**(A)** In the excised specimen of gastric tumor, a hard region of 5x7cm was observed 6cm from the distal margin. **(B)** The retinal tissue excised during the operation was 25x13x1.5cm in size. **(C–E)** Hematoxylin and eosin staining suggested poorly differentiated adenocarcinoma, infiltrating the whole gastric wall (T3), carcinoma infiltration in the nerve bundle, no tumor thrombus in the vascular, no cancer involvement in the distal resection margin, and no cancer metastasis in the omentum tissue. The lymph nodes in the greater curvature of the stomach showed cancer metastasis. **(F)** Programmed cell death ligand 1 (PD-L1) staining was negative.

A 65-year-old female was diagnosed with a gastric adenocarcinoma *via* chest and abdominal contrast-enhanced computed tomography (CT) and endoscopic biopsy in July 2017. After three courses of routine preoperative 5-fluorouracil/leucovorin, oxaliplatin, and docetaxel (FLOT) chemotherapy, she underwent radical gastrectomy. The final histopathology was that of a poorly differentiated adenocarcinoma with lymph node metastases (stage pT3N2) (**Figure 1**). The immunohistochemical findings were: Ki-67 (+15%), CDX2 (+), HER2 (-), CEA (+), CK7 (+), CK20 (-), AFP (-), and E-cadherin (+).

**Figure 2:**
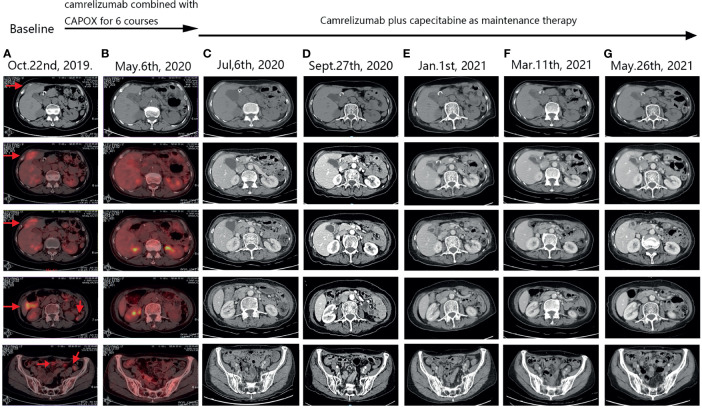
PET-CT and follow-up CT images. Arrows indicate tumors. **(A)** After the patient’s tumor recurred, pre-treatment positron emission tomography (PET)-computed tomography (CT) scan suggested metastatic lesions in the right lower chest wall, invasion of the adjacent peritoneum, and hepatic S4 involvement. Multiple patchy and nodular metastases in peritoneum of abdomen and pelvis. **(B)** After camrelizumab combined with CAPOX for 6 courses, positron emission tomography (PET)-computed tomography (CT) scan showed that the original metastatic lesions on the right lower chest wall disappeared, and the original multiple metastatic lesions on the peritoneum disappeared. No new tumors were observed in other organs. **(C–G)** During the period of camrelizumab plus capecitabine as maintenance therapy, regular CT scans suggested that the condition was stable, the original tumor lesions disappeared, and no new tumor lesions were found.

**Figure 3:**
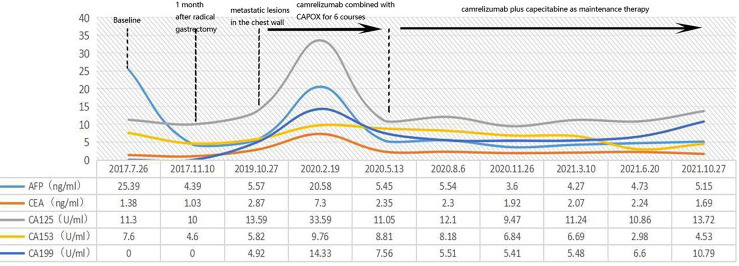
We closely followed the patient’s tumor markers, but unfortunately there were no significant abnormalities and no specific changes in the patient’s tumor markers during the onset and treatment.

After the completion of six courses of adjuvant FLOT chemotherapy, the patient was followed-up for nearly 2 years with no evidence of recurrence. In September 2019, she developed right lower abdominal pain. Positron emission tomography (PET)/CT revealed metabolically active soft tissue foci in the right lower chest wall that were thought to be metastases, with local invasion of the adjacent peritoneum, and suspected involvement of the liver. Multiple patches and nodular soft tissue foci in membranes were also thought to be metastases. The patient strongly desired immunotherapy, even though her PDL-1 expression status was unclear. After camrelizumab plus CAPOX (capecitabine plus oxaliplatin; six courses), PET/CT re-examination on May 6th, 2020, indicated the disappearance of all lesions in the right lower chest wall and the abdominal peritoneum. She thus exhibited a CCR. During the treatment, we tested the original stomach tumor again and the result was negative for PDL-1 (**Figure 1**). Based on the remarkable efficacy of camrelizumab plus CAPOX, we continued camrelizumab plus capecitabine maintenance therapy. To date, her quality of life is good, no new tumors have been detected on regular contrast-enhanced CT (**Figure 2**), and the symptom of right lower abdominal pain has disappeared completely(the NRS score was reduced from 8 to 0). In terms of adverse reactions, grade 1 haemangioma, grade 1 fatigue, grade 1 bone marrow hypocellular and grade 2 vomiting occurred during immunotherapy combined with chemotherapy, but it improved after symptomatic treatment. In addition, the patient developed grade 2 hypothyroidism that was identified as related to camrelizumab and was maintained on euthyroxine. The tumor markers of the patient were closely observed, and the changes were shown in **Figure 3**.

## Discussion

Systemic chemotherapy (fluoropyrimidine plus platinum) remains the principal first-line therapy for patients with unresectable advanced or metastatic gastric or gastroesophageal junction (G/GEJ) adenocarcinomas not expressing HER2 (7). However, increasing evidence suggests that immunotherapy and chemotherapy exert synergistic anti-tumour effects. The CheckMate 649 trial confirmed that nivolumab plus chemotherapy afforded significant clinical benefits in terms of overall survival (OS) and PFS in patients with PD-L1 combined positive scores of five or more, or one or more (5). Camrelizumab is a human immunoglobulin G4 monoclonal antibody developed in China; it restores the immune response by blocking the PD-1/PD-L1 pathway and improves the survival of patients with advanced tumours. One study from China found that camrelizumab plus CAPOX followed by camrelizumab plus apatinib afforded encouraging anti-tumour activity and manageable toxicity when employed as first-line therapy for patients with advanced or metastatic G/GEJ adenocarcinomas. The overall response rate was 58.3%. The median response duration was 5.7 months, the median OS 14.9 months, and the median PFS 6.8 months (6).

This is the first report of a CCR in a patient with an advanced gastric adenocarcinoma treated with camrelizumab plus chemotherapy followed by camrelizumab plus capecitabine first-line therapy. Her PD-L1 status remains unknown and her HER2 status is negative, she exhibited a significant response. Even if the patient is later found to be PD-L1-negative, her PFS has so far endured for more than 14 months. Treatment was (and is) well tolerated, she has evidenced only mild myelosuppression and immune system-related skin reactions. There are some reports of camrelizumab combined with lenvatinib (8). However, lenvatinib has not been recommended for the treatment of advanced gastric cancer in China, and lenvatinib may cause hemorrhages, which may be fatal to some patients with advanced gastric adenocarcinoma. Our new first-line treatment is different from all existing reports. This model facilitates the management of the whole process and provides a viable model that is tolerable and cost-effective. It may aid patients with PD-L1- and HER2-negative advanced gastric adenocarcinomas.

However, the study has shortcomings: Initially, patients with advanced gastric cancer can achieve a complete response of 1-4% with CAPOX or XELOX combination therapy, and the efficacy of the model of camrelizumab plus chemotherapy followed by camrelizumab plus capecitabine in advanced gastric cancer needs to be further verified through phase 2 and phase 3 clinical trials. Furthermore, the anti-tumor mechanism of camrelizumab combined with chemotherapy still needs further study. Finally, when the patient was found with chest wall metastasis, she did not consent to pathological biopsy. There was a lack of assessment of the possible tumor heterogeneity of the metastasis.

## Data Availability Statement

The original contributions presented in the study are included in the article/supplementary material. Further inquiries can be directed to the corresponding author.

## Ethics Statement

Written informed consent was obtained from the individual(s) for the publication of any potentially identifiable images or data included in this article.

## Author Contributions

JL, JY, and YC contributed to conception and design of the study. WW and XL organized the database. JL and JY wrote the first draft of the manuscript. All authors contributed to manuscript revision, read, and approved the submitted version.

## Conflict of Interest

The authors declare that the research was conducted in the absence of any commercial or financial relationships that could be construed as a potential conflict of interest.

## Publisher’s Note

All claims expressed in this article are solely those of the authors and do not necessarily represent those of their affiliated organizations, or those of the publisher, the editors and the reviewers. Any product that may be evaluated in this article, or claim that may be made by its manufacturer, is not guaranteed or endorsed by the publisher.
